# Workmen's Compensation Cases

**Published:** 1911-08-26

**Authors:** 


### Medico-Legal Points.

### WORKMEN'S COMPENSATION CASES.

### Is Frost-bite an Accident?

*Macdougall* v. *North British Railway Company*. —A case heard at Glasgow on June 3 raised the curious question whether a railway servant who suffers from frost-bite is the victim of an accident. It appeared that on February 14 last the pursuer was engaged during the night keeping clear the railway points, a task which was rendered necessary in consequence of the severity of the weather. While attending to his duties the fingers of both his hands were frost-bitten to such an extent as to incapacitate him from continuing at his usual occupation. So far as is known the point has not yet arisen or been decided whether injuries from frost-bite can " arise out of and in the course of a workman's employment," and can therefore be regarded as an accident under the Act.

Sheriff Davidson indicated that he had no hesitation in holding to the affirmative. Dr. Miller, of Fort William, medical referee for the district, having made a report on the case, an award was issued to the effect that the pursuer was entitled to compensation at the full rate of half wages up to the date of examination, and quarter wages thereafter.

### Appendicitis or Accident?

*Copsey* v. *Pattinson*.—In this case (Brampton, June 3) Walter Copsey died some time ago from the effect, it was alleged, of an accident while in the employment of the respondent. Evidence was given that he was partially dependent on his son, who was engaged by the respondent at £16 for the half-year. The chief point of interest in the case lay in the conflict of the medical evidence regarding the precise cause of death. Dr. Arnott, of Brampton, said he diagnosed an abscess and advised an operation the following day at the respondent's house. There was a large abscess and general peritonitis, the one being probably the result of the other. Death was due to exhaustion. Dr. Symington, also of Brampton, gave evidence to the same effect. Dr. Carrington Sykes and Dr. Ogilvy Ramsay gave it as their opinion, having heard the medical evidence, that the deceased died from appendicitis, which could not have been caused by strain.

His Honour, after consulting in private with Dr. Maclaren, of Carlisle, who sat with him as medical referee, said that this man had an accident which arose out of and in the course of his employment. He (the Judge) had been advised by the medical referee that the accident was, in fact, the cause of the death. As to the compensation, it was always difficult to arrive at it, but he awarded £100 and costs on C scale.

### A Divergence between Scotch and English Practice.

In *Mackay* v. *Rosie* (heard by all the Judges of the Court of Session, June 3) an important difference between the practice of the Scotch and English Courts was revealed. In England, where it has been found that a man has recovered from his injury, but that, on the medical evidence, it is probable that the trouble will break out later on, the practice has been to reduce the compensation to 1d. per week. In the case above mentioned respondent, while working on November 20, 1906, in the demolition of a tenement, was injured, and paid 18s. a week compensation for total incapacity. In July 1909 the appellant presented an application to have the payment reviewed, and the Sheriff-Substitute remitted to the medical referee for a report. In his reports the medical referee stated that Mackay was not incapacitated for work, but that while the ruptures caused by the accident did not at present prevent him from doing his ordinary work, yet they might and probably would become more marked and prove detrimental to him. In view of this report the Sheriff-Substitute held that Mackay had partially recovered his capacity, and diminished the weekly payment to 9s. per week. The Second Division heard the case and remitted it to the seven Judges, who decided that the Sheriff-Substitute's judgment was wrong, that the compensation must be ended. It was also held that the case should not be finally disposed of until it was seen whether the incapacity supervened.

Touching on the practice of the English Courts, the Lord President said that the practice of making a suspensory nominal award must either be sanctioned by the statute, or it must be bad. For this was a purely statutory matter to which there was no common law basis, and to which equitable considerations, even if existing, seemed to have no application. Until, therefore, the matter was otherwise settled by the House of Lords, his Lordship retained his opinion as stated in Clelland. That being so, it was, he thought, evident that there was no evidence at all to support the finding of the Sheriff-Substitute fixing the compensation at 9s. There was no proper course open to the Sheriff-Substitute but to end the compensation.

### Loss of an Eye.

In a case at Sheffield, May 26, it was made clear that the amount of compensation to be paid where a man has lost one eye must depend upon the kind of work he has been accustomed to do. Barker, who was employed as a machinist, had been in receipt of compensation which the employers sought to reduce on the ground that he could resume his old work. Medical evidence was adduced to the effect that there was no reason why Barker should not go back to his old work and earn his old rate of wages, which was 17s. 6d. per week. The workman denied that this was possible, for the work required the keenest of eyesight, the tools of the machine being adjusted to a sixty-fourth part of an inch. His Honour did not think the man could resume his ordinary work. It was not right to contend that the man, after losing one eye, could go on the labour market and earn his old rate of wages. The application was dismissed.

### Osteo-myelitis caused by Accident.

In a case at Rotherham, May 27, it appeared that the applicant was working at the Silverwood Colliery when his left wrist was crushed between two corves. He saw the company's doctor, and on February 8 he was admitted to the Rotherham Hospital, at which institution he had called once or twice previously as an out-patient. Various operations were performed on the limb while he was in the institution, and on March 3 amputation took place. In the medical testimony that was given it was shown that the disease from which the applicant had suffered was osteo-myelitis—the marrow of the bone was affected. For the applicant it was contended that this affection was due to the accident, a blow on the arm causing it to assert itself. The respondents produced an affidavit by Dr. Bell (late of the Rotherham Hospital), in whose care applicant had been, which considered that the organism had entered the system from a carbuncle between the shoulders. Judge Benson (who was assisted by Dr. Cuff as medical assessor) found for the applicant, the compensation being at the rate of 9s. 1d. per week.

